# Solvent inclusion in the crystal structure of bis­[(adamantan-1-yl)methanaminium chloride] 1,4-dioxane hemisolvate monohydrate explained using the computed crystal energy landscape

**DOI:** 10.1107/S2056989016013256

**Published:** 2016-08-26

**Authors:** Sharmarke Mohamed

**Affiliations:** a Khalifa University, PO BOX 127788, Abu Dhabi, United Arab Emirates

**Keywords:** crystal energy landscape, adamantanes, solvent-accessible voids, solvent inclusion

## Abstract

Repeated attempts to crystallize 1-adamantane­methyl­amine hydro­chloride as an anhydrate failed but the salt was successfully crystallized as a solvate (2C_11_H_20_N^+^·2Cl^−^·0.5C_4_H_8_O_2_·H_2_O), with water and 1,4-dioxane playing a structural role in the crystal and engaging in hydrogen-bonding inter­actions with the cation and anion. Computational crystal-structure prediction was used to rationalize the solvent-inclusion behaviour of this salt by computing the solvent-accessible voids in the predicted low-energy structures for the anhydrate: the global lattice-energy minimum structure, which has the same packing of the ions as the solvate, has solvent-accessible voids that account for 3.71% of the total unit-cell volume and is 6 kJ mol^−1^ more stable than the next most stable predicted structure.

## Abstract 2

Energy computations on the title salt show a clear preference for solvated structures, which correlates with the most effective formation of hydrogen bonds.

## Chemical context   

The rational synthesis of multi-component crystal forms using hydrogen-bond synthons (Desiraju, 1995 ▸) between donor and acceptor groups (Duggirala *et al.*, 2015 ▸) to direct the three-dimensional assembly of two or more mol­ecules in the solid state is an active area of crystal-engineering research. In recent years, there has been significant progress (Reilly *et al.*, 2016 ▸) in computational methods for predicting the most stable crystal structures of multi-component salt and co-crystal solid forms using only the mol­ecular structures as input. By comparison, the challenge of predicting when some mol­ecules will crystallize as solvates has received little attention (Braun *et al.*, 2013 ▸) from the crystal-engineering community and, despite evidence (Aakeröy *et al.*, 2007 ▸) from the Cambridge Structural Database (Groom *et al.*, 2016 ▸) that salt solid forms are more prone to crystallizing in structures with variable compositions and stoichiometries, the underlying factors behind the crystallization of salt solvates and the rational synthesis of such solid forms remains an under-explored area of crystal-engineering research. Previous work on the solvent-inclusion behaviour of substituted adamantane hydro­chloride salts (Mohamed *et al.*, 2016 ▸) has shown that mapping the percentage solvent-accessible volumes of predicted low-energy structures can provide a qualitative assessment of the likelihood of crystallizing non-stoichiometric channel hydrates of hydro­chloride salts. In this work, the computational model is extended to rationalize the solvent-inclusion behaviour of 1-adamantane­methyl­amine hydro­chloride on the basis of the packing efficiency of the ions and calculated solvent-accessible voids for the anhydrate.
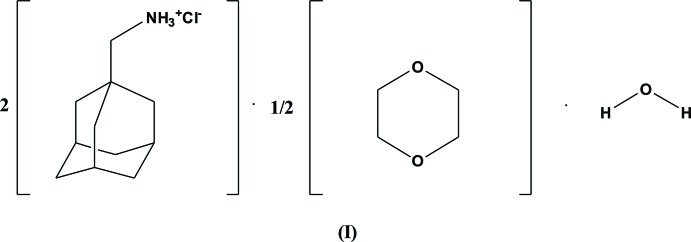


## Structural commentary   

**Figure 1:**
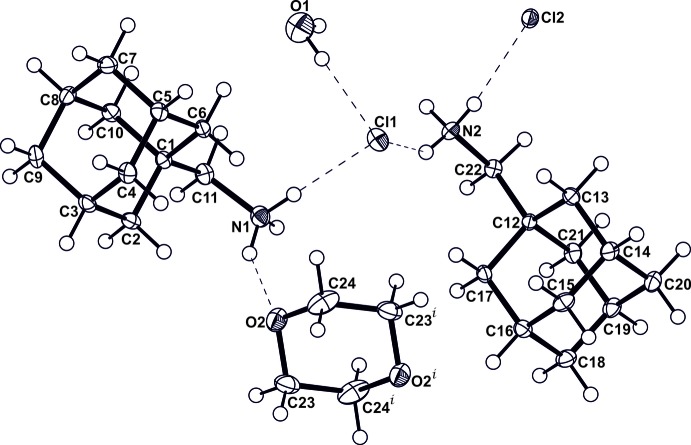
The mol­ecular structure of (I) with displacement ellipsoids drawn at the 50% probability level and hydrogen atoms are shown as spheres of arbitrary radius. Symmetry code: (i) 1 − *x*, 1 − *y*, 1 − *z*.

The asymmetric unit (Fig. 1 ▸) of the title structure (I) consists of two formula units of 1-adamantane­methyl­amine hydro­chloride, half a mol­ecule of 1,4-dioxane and one water mol­ecule. Both cations adopt a rigid conformation due to the adamantane ring and an overlay of the *ab initio* gas-phase-optimized conformation of the cation at the MP2/6-31G(d,p) level of theory with the experimental conformation of each symmetry-unique cation revealed a root-mean-squared deviation of less than 0.03 Å for the non-hydrogen atoms. The C1—C11—N1 and C12—C22—N2 bond angles for the cations are 113.76 (17) and 113.72 (16)°, which is consistent with the observation of identical mol­ecular conformations. The 1,4-dioxane mol­ecule lies on an inversion centre with C23—O2 and C24—O2 bond distances of 1.429 (3) and 1.425 (3) Å, respectively.

## Supra­molecular features   

**Figure 2:**
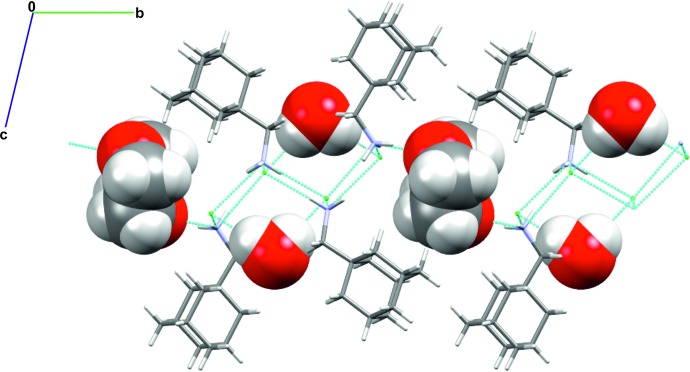
Crystal packing diagram for (I). The 1,4-dioxane and water mol­ecules are shown using a space-filling model. Inter­molecular hydrogen-bonding inter­actions are illustrated using blue dashed lines.

All N^+^—H bond lengths (Table 1 ▸) are between 0.85 and 0.98 Å and N^+^⋯Cl^−^ donor–acceptor distances are within the range 3.152–3.181 Å, which is consistent with the hydrogen-bond geometries in related 1-aminoadamantane hydro­chloride salts derived from primary amines such as adamantanamine hydro­chloride (Bélanger-Gariépy *et al.*, 1987 ▸) and (3,5-dimethyl-1-adamant­yl)ammonium chloride hydrate (Lou *et al.*, 2009 ▸). All N^+^—H hydrogen-bond donors on the cations engage in conventional hydrogen-bonding inter­actions with a chloride anion except for the N1^+^—H1*B*⋯O2 hydrogen bond which involves the O atom of 1,4-dioxane as a hydrogen-bond acceptor. All hydrogen-bonding inter­actions involving the donor–acceptor pairs N^+^⋯Cl^−^ or N^+^⋯O are characterized by discrete inter­actions of graph set *D* ^1^_1_(2). The crystal packing (Fig. 2 ▸) consists of a pleated ribbon stacking of the symmetry-inequivalent cations (*A* ^+^ and *B* ^+^) of 1-adamantane­methyl­amine along the *a* axis with a chloride ion hydrogen bonded to both symmetry-inequivalent cations in an infinite *A* ^+^⋯Cl^−^⋯*B* ^+^ pattern. This pleated ribbon stacking of the ions is similar to that observed in the crystal structure of 1-amino­adamantane hydro­chloride (Bélanger-Gariépy *et al.*, 1987 ▸). In the title structure, each water mol­ecule engages in discrete O—H⋯Cl^−^ hydrogen bonding inter­actions and each 1,4-dioxane mol­ecule acts as a hydrogen-bond acceptor to the N^+^—H donor of the cation. Both solvent mol­ecules occupy the voids between successive pleated ribbons formed from the stacking of hydrogen-bonded N^+^—H⋯Cl^−^ charged units.

## Computed crystal energy landscape   

**Figure 3:**
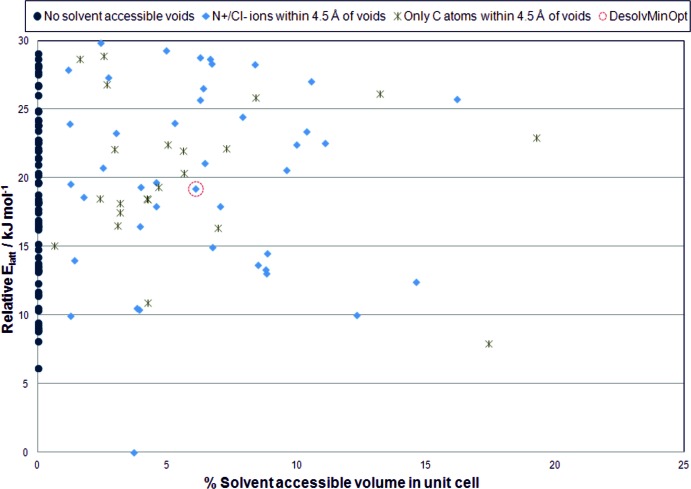
Predicted crystal energy landscape of (adamantan-1-yl)methanaminium chloride. The lattice energy is plotted relative to the predicted global minimum structure for the salt. The data point labelled *DesolvMinOpt* corresponds to the theoretical lattice energy minimum structure that would result from desolvation of the experimental (adamantan-1-yl)methanaminium chloride 1,4-dioxane hydrate structure.

The computed crystal energy landscape (Fig. 3 ▸) of 1-adamantane­methyl­amine hydro­chloride was used to assess the possibility of solvent inclusion for this salt by estimating the percentage solvent-accessible volume in the predicted most stable packings. The most stable structure on the crystal energy landscape of the anhydrate displays a total potential solvent-accessible volume of 45.6 Å^3^, which corresponds to 3.71% of the unit-cell volume. Assuming that each water mol­ecule occupies an approximate total volume of 40 Å^3^, this would suggest that the global minimum structure could be crystallized by dehydration of a monohydrate of the salt. The global lattice energy minimum structure is approximately 6 kJ mol^−1^ more stable than the nearest competing second-ranked structure. The observation of a clearly preferred global lattice energy minimum structure with solvent-accessible voids is not conclusive in suggesting that this hydro­chloride salt cannot be crystallized as an anhydrate, but it does suggest that this system will have difficulties crystallizing as an anhydrate since there is an energetic preference for a packing of the ions that is susceptible to solvent inclusion. Although the second-ranked most stable predicted structure does not have any solvent-accessible voids, this structure is energetically competitive with the third-ranked structure which displays an unusually large percentage solvent-accessible volume of 17.42% of the unit cell. The majority of the predicted structures within 10 kJ mol^−1^ of the global minimum structure that have solvent-accessible voids have crystal voids that are located within 4.5 Å of the charged N^+^/Cl^−^ ions, which is consistent with the observation that both the water and 1,4-dioxane solvent mol­ecules in the experimental structure engage in hydrogen-bonding inter­actions with the N^+^—H donor and Cl^−^ acceptor groups of the salt.

Although there is a clear thermodynamically preferred global minimum structure with solvent-accessible voids, the calculations also reveal that there are a number of energetically competitive packings of the ions within 10 kJ mol^−1^ of the global minimum structure that do not have solvent-accessible voids. However, 88% of these structures have one or two unused N^+^—H donors as judged by N^+^⋯Cl^−^ distances that are longer than the sum of the van der Waals radii of the N and Cl atoms, suggesting challenges in close packing of the ions which is consistent with the observation of solvent inclusion in this salt with solvent mol­ecules engaged in hydrogen-bonding inter­actions. The structurally related 1-amino­adamantane mol­ecule, which differs from 1-adamantanemethlyamine in that it lacks a methyl­ene group bridging the adamantane ring and NH_2_ functional group displays a crystal energy landscape (Mohamed *et al.*, 2016 ▸) with a single preferred global minimum structure corresponding to the experimentally observed anhydrate structure of the salt. This illustrates the sensitivity of crystal packing to minor modifications in mol­ecular structure and the value of mapping the percentage solvent-accessible voids in predicted low-energy structures of hydro­chlorides as a means for assessing the possibility of solvent inclusion.

## Database survey   

A search of the Cambridge Structural Database (CSD Version 5.37 plus three updates, no filters; Groom *et al.* 2016 ▸) has shown that there are no previously reported crystal structures of 1-adamantane­methyl­amine or multi-component salt or co-crystal structures of this primary amine. However, a di­methyl­formamide solvate of a platinum coordination complex involving two crystallographically inequivalent 1-adamant­ane­methyl­amine mol­ecules coordinated onto platinum(II) metal has been reported (DUHKAT: Rochon & Tessier, 2009 ▸) from crystallization experiments involving *cisplatin* [*cis*-Pt(NH_3_)_2_Cl_2_] and 1-adamantane­methyl­amine in di­methyl­formamide. There are a number of reported crystal structures of substituted amino­adamantane hydro­chloride salts such as 1-aminoadamantanamine hydro­chloride (FINVAZ: Bélanger-Gariépy *et al.*, 1987 ▸), (3,5-dimethyl-1-adamant­yl)ammonium chloride hydrate (DUCYAC: Lou *et al.*, 2009 ▸) and (*RS*)-1-(1-adamant­yl)ethanamine hydro­chloride (TOKWUN: Mishnev & Stepanovs, 2014 ▸).

## Synthesis and crystallization   

A 1:5 ratio of HCl:acetone mixture was prepared and 0.3 ml of 1-adamantane­methyl­amine was added to a vial containing 2 ml of the HCl:acetone mixture. The contents of the vial were further diluted by adding 3 ml of a 1:1 mixture of 1,4-dioxane:ethanol. The contents of the vial were shaken vigorously for two minutes and filtered under gravity. The solvent was allowed to evaporate under laboratory temperature and pressure conditions and after 24 h crystals of the title solvate with needle morphology were isolated.

## Refinement   

Crystal data, data collection and structure refinement details are summarized in Table 2 ▸. Hydrogen atoms attached to N and O atoms were located from difference Fourier maps and freely refined. All other hydrogen atoms were positioned geom­etrically (C—H = 0.99–1.00 Å) and refined using a riding model with *U*_iso_(H) = 1.2*U*_eq_(carrier).

## Computational modelling methodology   

The crystal energy landscape of 1-adamantane­methyl­amine hydro­chloride was calculated using a search criterion that restricted crystal packings to those with one (*Z*′ = 1) or two (*Z*′ = 2) formula units of the ions in the asymmetric unit using the *Materials Studio 8.0* (Accelrys, 2014 ▸) code. Hypothetical crystal structures were generated in five of the most common space groups (*P* 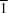, *P*2_1_, *P*2_1_/*c*, *P*2_1_2_1_2_1_, *C*2/*c*) for organic crystal structures using the MP2/6-31G(d,p) optimized geometry for the protonated 1-adamantane­methyl­amine cation. The atomic charges on the cation were derived by fitting to the mol­ecular electrostatic potential of the optimized conformation using the ChelpG (Breneman & Wiberg, 1990 ▸) scheme. The mol­ecular geometry and fitted charges for the cation were calculated using *GAUSSIAN09* (Frisch *et al.*, 2009 ▸). The final lattice energies for the predicted structures were estimated using a distributed multipole model for the charges using *DMACRYS* (Price *et al.*, 2010 ▸). Dispersion-repulsion contributions towards the lattice energy were estimated using the revised Williams99 force field (Williams, 2001 ▸) supplemented with the potential parameter set for the Cl^−^ ion (Hejczyk, 2010 ▸). For all predicted structures in the crystal energy landscape, the solvent-accessible volume per unit cell was estimated using *PLATON* (Spek, 2009 ▸) assuming a probe radius of 1.2 Å. Detailed settings for the *Materials Studio 8.0* search for putative crystal structures and the *DMACRYS* lattice energy optimizations are the same as those reported in recent work (Mohamed *et al.*, 2016 ▸) investigating the utility of computed crystal energy landscapes for inferring the risk of crystal hydration in substituted adamantane hydro­chloride salts.

## Supplementary Material

Crystal structure: contains datablock(s) I. DOI: 10.1107/S2056989016013256/hb7608sup1.cif

Structure factors: contains datablock(s) I. DOI: 10.1107/S2056989016013256/hb7608Isup2.hkl

Supporting information file. DOI: 10.1107/S2056989016013256/hb7608Isup4.cdx

CCDC reference: 1499645

Additional supporting information: crystallographic information; 3D view; checkCIF report

## Figures and Tables

**Table 1:** Hydrogen-bond geometry (Å, °)

*D*—H⋯*A*	*D*—H	H⋯*A*	*D*⋯*A*	*D*—H⋯*A*
O1—H1*D*⋯Cl2^i^	0.82 (3)	2.48 (3)	3.295 (2)	175 (3)
O1—H1*E*⋯Cl1	0.91 (4)	2.36 (4)	3.265 (2)	180 (3)
N1—H1*A*⋯Cl2^ii^	0.85 (3)	2.33 (3)	3.161 (2)	163 (2)
N1—H1*B*⋯O2	0.89 (3)	2.10 (3)	2.867 (3)	144 (2)
N1—H1*C*⋯Cl1	0.98 (3)	2.19 (3)	3.152 (2)	166 (2)
N2—H2*C*⋯Cl2^i^	0.86 (2)	2.31 (3)	3.166 (2)	172 (2)
N2—H2*D*⋯Cl1	0.86 (3)	2.48 (3)	3.171 (2)	138 (2)
N2—H2*E*⋯Cl2	0.90 (3)	2.30 (3)	3.181 (2)	166 (2)

Symmetry codes: (i) 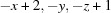; (ii) 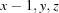.

**Table 2:** Experimental details

Crystal data	
Chemical formula	2C_11_H_20_N^+^·2Cl^−^·0.5C_4_H_8_O_2_·H_2_O
*M*_r_	465.53
Crystal system, space group	Triclinic, *P* 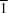
Temperature (K)	100
*a*, *b*, *c* (Å)	6.4941 (11), 13.491 (2), 15.086 (3)
α, β, γ (°)	102.911 (3), 91.824 (3), 101.500 (3)
*V* (Å^3^)	1258.5 (4)
*Z*	2
Radiation type	Mo *K*α
μ (mm^−1^)	0.28
Crystal size (mm)	0.2 × 0.05 × 0.05

Data collection	
Diffractometer	Bruker APEXII CCD
Absorption correction	Multi-scan (*SADABS*; Bruker, 2015 ▸)
*T*_min_, *T*_max_	0.655, 0.746
No. of measured, independent and observed [*I* > 2σ(*I*)] reflections	35014, 6312, 4285
*R*_int_	0.096
(sin θ/λ)_max_ (Å^−1^)	0.670

Refinement	
*R*[*F* ^2^ > 2σ(*F* ^2^)], *wR*(*F* ^2^), *S*	0.056, 0.105, 1.06
No. of reflections	6312
No. of parameters	303
H-atom treatment	H atoms treated by a mixture of independent and constrained refinement
Δρ_max_, Δρ_min_ (e Å^−3^)	0.49, −0.39

Computer programs: *APEX2* and *SAINT* (Bruker 2015 ▸), *XT* (Sheldrick, 2015 ▸), *SHELXL* (Sheldrick, 2008 ▸) and *OLEX2* (Dolomanov *et al.*, 2009 ▸).

## supplementary crystallographic information

### Crystal data

**Table:**

2C_11_H_20_N^+^·2Cl^−^·0.5C_4_H_8_O_2_·H_2_O	*Z* = 2
*M**_r_* = 465.53	*F*(000) = 508
Triclinic, *P*1	*D*_x_ = 1.229 Mg m^−^^3^
*a* = 6.4941 (11) Å	Mo *K*α radiation, λ = 0.71073 Å
*b* = 13.491 (2) Å	Cell parameters from 4531 reflections
*c* = 15.086 (3) Å	θ = 2.3–28.0°
α = 102.911 (3)°	µ = 0.28 mm^−^^1^
β = 91.824 (3)°	*T* = 100 K
γ = 101.500 (3)°	Needle, clear light colourless
*V* = 1258.5 (4) Å^3^	0.2 × 0.05 × 0.05 mm

### Data collection

**Table:**

Bruker APEXII CCD diffractometer	4285 reflections with *I* > 2σ(*I*)
φ and ω scans	*R*_int_ = 0.096
Absorption correction: multi-scan (SADABS; Bruker, 2015)	θ_max_ = 28.4°, θ_min_ = 1.6°
*T*_min_ = 0.655, *T*_max_ = 0.746	*h* = −8→8
35014 measured reflections	*k* = −18→17
6312 independent reflections	*l* = −20→20

### Refinement

**Table:**

Refinement on *F*^2^	0 restraints
Least-squares matrix: full	Hydrogen site location: mixed
*R*[*F*^2^ > 2σ(*F*^2^)] = 0.056	H atoms treated by a mixture of independent and constrained refinement
*wR*(*F*^2^) = 0.105	*w* = 1/[σ^2^(*F*_o_^2^) + (0.0415*P*)^2^ + 0.1332*P*] where *P* = (*F*_o_^2^ + 2*F*_c_^2^)/3
*S* = 1.06	(Δ/σ)_max_ = 0.001
6312 reflections	Δρ_max_ = 0.49 e Å^−^^3^
303 parameters	Δρ_min_ = −0.39 e Å^−^^3^

### Special details

**Table:**

Geometry. All esds (except the esd in the dihedral angle between two l.s. planes) are estimated using the full covariance matrix. The cell esds are taken into account individually in the estimation of esds in distances, angles and torsion angles; correlations between esds in cell parameters are only used when they are defined by crystal symmetry. An approximate (isotropic) treatment of cell esds is used for estimating esds involving l.s. planes.

### Fractional atomic coordinates and isotropic or equivalent isotropic displacement parameters (Å^2^)

**Table:**

	*x*	*y*	*z*	*U*_iso_*/*U*_eq_	
Cl1	0.87597 (8)	0.25420 (4)	0.41712 (4)	0.02117 (14)	
O1	0.7543 (3)	0.01392 (17)	0.29766 (13)	0.0350 (4)	
H1D	0.744 (5)	−0.019 (3)	0.337 (2)	0.067 (12)*	
H1E	0.789 (5)	0.081 (3)	0.331 (2)	0.069 (11)*	
N1	0.3859 (3)	0.22378 (16)	0.37519 (13)	0.0184 (4)	
H1A	0.347 (4)	0.202 (2)	0.4221 (18)	0.034 (7)*	
H1B	0.331 (4)	0.280 (2)	0.3786 (16)	0.032 (7)*	
H1C	0.540 (5)	0.242 (2)	0.3820 (18)	0.049 (8)*	
C1	0.3307 (3)	0.17360 (15)	0.20310 (13)	0.0124 (4)	
C2	0.1981 (3)	0.25504 (15)	0.19496 (14)	0.0149 (4)	
H2A	0.0482	0.2266	0.2017	0.018*	
H2B	0.2472	0.3185	0.2443	0.018*	
C3	0.2196 (3)	0.28259 (15)	0.10183 (14)	0.0150 (4)	
H3	0.1337	0.3354	0.0972	0.018*	
C4	0.4520 (3)	0.32784 (16)	0.09241 (14)	0.0161 (4)	
H4A	0.4666	0.3470	0.0329	0.019*	
H4B	0.5031	0.3914	0.1414	0.019*	
C5	0.5838 (3)	0.24691 (15)	0.09893 (13)	0.0141 (4)	
H5	0.7351	0.2763	0.0925	0.017*	
C6	0.5625 (3)	0.21869 (16)	0.19195 (13)	0.0131 (4)	
H6A	0.6486	0.1669	0.1966	0.016*	
H6B	0.6157	0.2816	0.2414	0.016*	
C7	0.5040 (3)	0.14874 (16)	0.02254 (14)	0.0172 (4)	
H7A	0.5184	0.1662	−0.0376	0.021*	
H7B	0.5894	0.0964	0.0261	0.021*	
C8	0.2722 (3)	0.10384 (16)	0.03253 (14)	0.0160 (4)	
H8	0.2205	0.0399	−0.0172	0.019*	
C9	0.1409 (3)	0.18475 (16)	0.02568 (14)	0.0174 (4)	
H9A	0.1532	0.2025	−0.0344	0.021*	
H9B	−0.0094	0.1558	0.0312	0.021*	
C10	0.2512 (3)	0.07589 (15)	0.12521 (13)	0.0153 (4)	
H10A	0.3343	0.0228	0.1293	0.018*	
H10B	0.1017	0.0461	0.1314	0.018*	
C11	0.3041 (3)	0.13900 (16)	0.29224 (13)	0.0166 (4)	
H11A	0.1525	0.1115	0.2965	0.020*	
H11B	0.3785	0.0815	0.2912	0.020*	
Cl2	1.31606 (7)	0.11138 (4)	0.53791 (3)	0.01540 (12)	
O2	0.4015 (2)	0.44325 (11)	0.41377 (10)	0.0234 (4)	
C23	0.2855 (3)	0.49326 (18)	0.48258 (16)	0.0282 (6)	
H23A	0.1386	0.4529	0.4765	0.034*	
H23B	0.2817	0.5637	0.4744	0.034*	
C24	0.6155 (4)	0.49844 (19)	0.42468 (16)	0.0307 (6)	
H24A	0.6217	0.5691	0.4150	0.037*	
H24B	0.6960	0.4621	0.3781	0.037*	
N2	0.8388 (3)	0.12390 (16)	0.56887 (12)	0.0161 (4)	
H2C	0.785 (4)	0.062 (2)	0.5387 (16)	0.022 (6)*	
H2D	0.788 (4)	0.165 (2)	0.5421 (17)	0.031 (7)*	
H2E	0.977 (4)	0.1327 (19)	0.5613 (16)	0.033 (7)*	
C12	0.8559 (3)	0.24495 (15)	0.72436 (13)	0.0113 (4)	
C13	1.0863 (3)	0.29809 (15)	0.71971 (14)	0.0145 (4)	
H13A	1.1811	0.2563	0.7388	0.017*	
H13B	1.1111	0.3024	0.6561	0.017*	
C14	1.1355 (3)	0.40791 (16)	0.78235 (14)	0.0157 (4)	
H14	1.2854	0.4421	0.7784	0.019*	
C15	0.9883 (3)	0.47258 (16)	0.75293 (14)	0.0172 (4)	
H15A	1.0211	0.5437	0.7928	0.021*	
H15B	1.0101	0.4781	0.6894	0.021*	
C16	0.7587 (3)	0.42114 (16)	0.75939 (13)	0.0154 (4)	
H16	0.6633	0.4637	0.7403	0.019*	
C17	0.7099 (3)	0.31176 (15)	0.69597 (13)	0.0131 (4)	
H17A	0.7303	0.3170	0.6323	0.016*	
H17B	0.5610	0.2781	0.6989	0.016*	
C18	0.7244 (3)	0.41275 (16)	0.85783 (13)	0.0173 (4)	
H18A	0.7535	0.4831	0.8989	0.021*	
H18B	0.5760	0.3791	0.8619	0.021*	
C19	0.8716 (3)	0.34845 (16)	0.88730 (14)	0.0167 (4)	
H19	0.8498	0.3433	0.9516	0.020*	
C20	1.1009 (3)	0.40095 (16)	0.88100 (14)	0.0181 (5)	
H20A	1.1967	0.3603	0.9011	0.022*	
H20B	1.1332	0.4716	0.9216	0.022*	
C21	0.8212 (3)	0.23899 (16)	0.82383 (13)	0.0148 (4)	
H21A	0.6729	0.2053	0.8280	0.018*	
H21B	0.9132	0.1961	0.8434	0.018*	
C22	0.8034 (3)	0.13331 (15)	0.66746 (13)	0.0135 (4)	
H22A	0.8908	0.0923	0.6928	0.016*	
H22B	0.6538	0.1027	0.6727	0.016*	

### Atomic displacement parameters (Å^2^)

**Table:**

	*U*^11^	*U*^22^	*U*^33^	*U*^12^	*U*^13^	*U*^23^
Cl1	0.0178 (3)	0.0247 (3)	0.0229 (3)	0.0049 (2)	0.0017 (2)	0.0091 (2)
O1	0.0399 (11)	0.0353 (12)	0.0310 (11)	0.0050 (9)	0.0045 (9)	0.0126 (10)
N1	0.0183 (10)	0.0238 (11)	0.0151 (10)	0.0056 (9)	0.0019 (8)	0.0071 (8)
C1	0.0111 (9)	0.0120 (10)	0.0140 (10)	0.0014 (8)	0.0006 (8)	0.0037 (8)
C2	0.0119 (10)	0.0136 (10)	0.0197 (11)	0.0041 (8)	0.0023 (8)	0.0035 (9)
C3	0.0145 (10)	0.0123 (10)	0.0194 (11)	0.0052 (8)	−0.0013 (8)	0.0043 (8)
C4	0.0187 (11)	0.0146 (11)	0.0162 (11)	0.0026 (9)	−0.0006 (9)	0.0070 (9)
C5	0.0100 (10)	0.0165 (11)	0.0152 (10)	0.0008 (8)	0.0025 (8)	0.0045 (8)
C6	0.0111 (10)	0.0144 (10)	0.0138 (10)	0.0029 (8)	−0.0010 (8)	0.0034 (8)
C7	0.0189 (11)	0.0192 (11)	0.0150 (11)	0.0079 (9)	0.0034 (9)	0.0037 (9)
C8	0.0179 (11)	0.0142 (11)	0.0137 (10)	0.0027 (9)	−0.0021 (8)	−0.0002 (8)
C9	0.0146 (10)	0.0188 (11)	0.0179 (11)	0.0013 (9)	−0.0054 (8)	0.0052 (9)
C10	0.0143 (10)	0.0107 (10)	0.0204 (11)	0.0019 (8)	−0.0006 (8)	0.0036 (8)
C11	0.0140 (10)	0.0172 (11)	0.0183 (11)	0.0007 (9)	0.0006 (8)	0.0061 (9)
Cl2	0.0146 (2)	0.0168 (3)	0.0135 (2)	0.0022 (2)	−0.00028 (19)	0.00194 (19)
O2	0.0235 (8)	0.0231 (9)	0.0205 (8)	0.0020 (7)	−0.0008 (7)	0.0018 (7)
C23	0.0159 (11)	0.0221 (13)	0.0420 (15)	0.0058 (10)	0.0027 (10)	−0.0034 (11)
C24	0.0343 (14)	0.0258 (13)	0.0270 (13)	−0.0027 (11)	0.0181 (11)	0.0018 (10)
N2	0.0175 (10)	0.0147 (10)	0.0136 (9)	0.0033 (8)	−0.0004 (8)	−0.0013 (8)
C12	0.0111 (9)	0.0121 (10)	0.0102 (9)	0.0019 (8)	−0.0001 (8)	0.0019 (8)
C13	0.0103 (10)	0.0169 (11)	0.0155 (10)	0.0023 (8)	0.0013 (8)	0.0025 (8)
C14	0.0114 (10)	0.0133 (10)	0.0189 (11)	−0.0015 (8)	0.0018 (8)	0.0001 (8)
C15	0.0226 (11)	0.0119 (11)	0.0163 (11)	0.0024 (9)	0.0033 (9)	0.0026 (8)
C16	0.0157 (10)	0.0163 (11)	0.0155 (11)	0.0057 (9)	0.0012 (8)	0.0040 (8)
C17	0.0117 (10)	0.0170 (11)	0.0115 (10)	0.0044 (8)	−0.0002 (8)	0.0042 (8)
C18	0.0173 (11)	0.0182 (11)	0.0153 (11)	0.0041 (9)	0.0042 (8)	0.0010 (9)
C19	0.0195 (11)	0.0201 (11)	0.0103 (10)	0.0038 (9)	0.0023 (8)	0.0033 (8)
C20	0.0186 (11)	0.0169 (11)	0.0158 (11)	0.0019 (9)	−0.0032 (9)	0.0000 (9)
C21	0.0152 (10)	0.0172 (11)	0.0130 (10)	0.0022 (9)	0.0013 (8)	0.0065 (8)
C22	0.0133 (10)	0.0129 (10)	0.0139 (10)	0.0013 (8)	0.0013 (8)	0.0039 (8)

### Geometric parameters (Å, º)

**Table:**

O1—H1D	0.82 (3)	C23—H23B	0.9900
O1—H1E	0.91 (4)	C23—C24^i^	1.493 (3)
N1—H1A	0.85 (3)	C24—C23^i^	1.493 (3)
N1—H1B	0.89 (3)	C24—H24A	0.9900
N1—H1C	0.98 (3)	C24—H24B	0.9900
N1—C11	1.494 (3)	N2—H2C	0.86 (2)
C1—C2	1.547 (3)	N2—H2D	0.86 (3)
C1—C6	1.537 (3)	N2—H2E	0.90 (3)
C1—C10	1.544 (3)	N2—C22	1.493 (3)
C1—C11	1.523 (3)	C12—C13	1.536 (3)
C2—H2A	0.9900	C12—C17	1.543 (3)
C2—H2B	0.9900	C12—C21	1.542 (3)
C2—C3	1.535 (3)	C12—C22	1.523 (3)
C3—H3	1.0000	C13—H13A	0.9900
C3—C4	1.535 (3)	C13—H13B	0.9900
C3—C9	1.529 (3)	C13—C14	1.535 (3)
C4—H4A	0.9900	C14—H14	1.0000
C4—H4B	0.9900	C14—C15	1.533 (3)
C4—C5	1.533 (3)	C14—C20	1.533 (3)
C5—H5	1.0000	C15—H15A	0.9900
C5—C6	1.537 (3)	C15—H15B	0.9900
C5—C7	1.535 (3)	C15—C16	1.530 (3)
C6—H6A	0.9900	C16—H16	1.0000
C6—H6B	0.9900	C16—C17	1.535 (3)
C7—H7A	0.9900	C16—C18	1.534 (3)
C7—H7B	0.9900	C17—H17A	0.9900
C7—C8	1.532 (3)	C17—H17B	0.9900
C8—H8	1.0000	C18—H18A	0.9900
C8—C9	1.532 (3)	C18—H18B	0.9900
C8—C10	1.531 (3)	C18—C19	1.530 (3)
C9—H9A	0.9900	C19—H19	1.0000
C9—H9B	0.9900	C19—C20	1.531 (3)
C10—H10A	0.9900	C19—C21	1.536 (3)
C10—H10B	0.9900	C20—H20A	0.9900
C11—H11A	0.9900	C20—H20B	0.9900
C11—H11B	0.9900	C21—H21A	0.9900
O2—C23	1.429 (3)	C21—H21B	0.9900
O2—C24	1.425 (3)	C22—H22A	0.9900
C23—H23A	0.9900	C22—H22B	0.9900
			
H1D—O1—H1E	102 (3)	C24^i^—C23—H23B	109.5
H1A—N1—H1B	105 (2)	O2—C24—C23^i^	111.48 (18)
H1A—N1—H1C	106 (2)	O2—C24—H24A	109.3
H1B—N1—H1C	111 (2)	O2—C24—H24B	109.3
C11—N1—H1A	108.3 (17)	C23^i^—C24—H24A	109.3
C11—N1—H1B	113.6 (16)	C23^i^—C24—H24B	109.3
C11—N1—H1C	112.3 (16)	H24A—C24—H24B	108.0
C6—C1—C2	108.98 (16)	H2C—N2—H2D	106 (2)
C6—C1—C10	108.68 (16)	H2C—N2—H2E	105 (2)
C10—C1—C2	108.41 (16)	H2D—N2—H2E	108 (2)
C11—C1—C2	111.88 (16)	C22—N2—H2C	109.5 (15)
C11—C1—C6	111.96 (16)	C22—N2—H2D	116.8 (16)
C11—C1—C10	106.81 (16)	C22—N2—H2E	110.1 (16)
C1—C2—H2A	109.7	C13—C12—C17	109.06 (16)
C1—C2—H2B	109.7	C13—C12—C21	108.64 (16)
H2A—C2—H2B	108.2	C21—C12—C17	108.24 (15)
C3—C2—C1	109.98 (15)	C22—C12—C13	112.02 (15)
C3—C2—H2A	109.7	C22—C12—C17	112.31 (16)
C3—C2—H2B	109.7	C22—C12—C21	106.42 (15)
C2—C3—H3	109.4	C12—C13—H13A	109.6
C4—C3—C2	109.32 (16)	C12—C13—H13B	109.6
C4—C3—H3	109.4	H13A—C13—H13B	108.1
C9—C3—C2	109.66 (16)	C14—C13—C12	110.21 (15)
C9—C3—H3	109.4	C14—C13—H13A	109.6
C9—C3—C4	109.58 (16)	C14—C13—H13B	109.6
C3—C4—H4A	109.8	C13—C14—H14	109.6
C3—C4—H4B	109.8	C15—C14—C13	109.61 (16)
H4A—C4—H4B	108.2	C15—C14—H14	109.6
C5—C4—C3	109.55 (16)	C20—C14—C13	109.53 (17)
C5—C4—H4A	109.8	C20—C14—H14	109.6
C5—C4—H4B	109.8	C20—C14—C15	108.95 (16)
C4—C5—H5	109.5	C14—C15—H15A	109.7
C4—C5—C6	109.41 (15)	C14—C15—H15B	109.7
C4—C5—C7	109.42 (16)	H15A—C15—H15B	108.2
C6—C5—H5	109.5	C16—C15—C14	109.89 (16)
C7—C5—H5	109.5	C16—C15—H15A	109.7
C7—C5—C6	109.35 (16)	C16—C15—H15B	109.7
C1—C6—C5	110.25 (16)	C15—C16—H16	109.6
C1—C6—H6A	109.6	C15—C16—C17	108.88 (16)
C1—C6—H6B	109.6	C15—C16—C18	109.79 (17)
C5—C6—H6A	109.6	C17—C16—H16	109.6
C5—C6—H6B	109.6	C18—C16—H16	109.6
H6A—C6—H6B	108.1	C18—C16—C17	109.39 (16)
C5—C7—H7A	109.8	C12—C17—H17A	109.6
C5—C7—H7B	109.8	C12—C17—H17B	109.6
H7A—C7—H7B	108.2	C16—C17—C12	110.35 (16)
C8—C7—C5	109.49 (16)	C16—C17—H17A	109.6
C8—C7—H7A	109.8	C16—C17—H17B	109.6
C8—C7—H7B	109.8	H17A—C17—H17B	108.1
C7—C8—H8	109.4	C16—C18—H18A	109.8
C9—C8—C7	109.42 (17)	C16—C18—H18B	109.8
C9—C8—H8	109.4	H18A—C18—H18B	108.2
C10—C8—C7	109.55 (16)	C19—C18—C16	109.57 (16)
C10—C8—H8	109.4	C19—C18—H18A	109.8
C10—C8—C9	109.59 (16)	C19—C18—H18B	109.8
C3—C9—C8	109.52 (16)	C18—C19—H19	109.6
C3—C9—H9A	109.8	C18—C19—C20	109.50 (17)
C3—C9—H9B	109.8	C18—C19—C21	108.92 (17)
C8—C9—H9A	109.8	C20—C19—H19	109.6
C8—C9—H9B	109.8	C20—C19—C21	109.70 (16)
H9A—C9—H9B	108.2	C21—C19—H19	109.6
C1—C10—H10A	109.6	C14—C20—H20A	109.7
C1—C10—H10B	109.6	C14—C20—H20B	109.7
C8—C10—C1	110.29 (16)	C19—C20—C14	109.72 (16)
C8—C10—H10A	109.6	C19—C20—H20A	109.7
C8—C10—H10B	109.6	C19—C20—H20B	109.7
H10A—C10—H10B	108.1	H20A—C20—H20B	108.2
N1—C11—C1	113.76 (17)	C12—C21—H21A	109.6
N1—C11—H11A	108.8	C12—C21—H21B	109.6
N1—C11—H11B	108.8	C19—C21—C12	110.50 (16)
C1—C11—H11A	108.8	C19—C21—H21A	109.6
C1—C11—H11B	108.8	C19—C21—H21B	109.6
H11A—C11—H11B	107.7	H21A—C21—H21B	108.1
C24—O2—C23	109.78 (16)	N2—C22—C12	113.72 (16)
O2—C23—H23A	109.5	N2—C22—H22A	108.8
O2—C23—H23B	109.5	N2—C22—H22B	108.8
O2—C23—C24^i^	110.56 (18)	C12—C22—H22A	108.8
H23A—C23—H23B	108.1	C12—C22—H22B	108.8
C24^i^—C23—H23A	109.5	H22A—C22—H22B	107.7
			
C1—C2—C3—C4	−60.0 (2)	C24—O2—C23—C24^i^	−56.6 (3)
C1—C2—C3—C9	60.1 (2)	C12—C13—C14—C15	−59.2 (2)
C2—C1—C6—C5	−58.9 (2)	C12—C13—C14—C20	60.3 (2)
C2—C1—C10—C8	59.2 (2)	C13—C12—C17—C16	−59.1 (2)
C2—C1—C11—N1	−64.9 (2)	C13—C12—C21—C19	58.9 (2)
C2—C3—C4—C5	60.5 (2)	C13—C12—C22—N2	−58.5 (2)
C2—C3—C9—C8	−60.0 (2)	C13—C14—C15—C16	60.1 (2)
C3—C4—C5—C6	−60.2 (2)	C13—C14—C20—C19	−59.6 (2)
C3—C4—C5—C7	59.6 (2)	C14—C15—C16—C17	−60.3 (2)
C4—C3—C9—C8	60.0 (2)	C14—C15—C16—C18	59.5 (2)
C4—C5—C6—C1	59.8 (2)	C15—C14—C20—C19	60.3 (2)
C4—C5—C7—C8	−59.9 (2)	C15—C16—C17—C12	60.1 (2)
C5—C7—C8—C9	60.2 (2)	C15—C16—C18—C19	−59.1 (2)
C5—C7—C8—C10	−60.0 (2)	C16—C18—C19—C20	59.5 (2)
C6—C1—C2—C3	59.0 (2)	C16—C18—C19—C21	−60.4 (2)
C6—C1—C10—C8	−59.1 (2)	C17—C12—C13—C14	58.4 (2)
C6—C1—C11—N1	57.8 (2)	C17—C12—C21—C19	−59.4 (2)
C6—C5—C7—C8	59.9 (2)	C17—C12—C22—N2	64.6 (2)
C7—C5—C6—C1	−60.0 (2)	C17—C16—C18—C19	60.3 (2)
C7—C8—C9—C3	−60.2 (2)	C18—C16—C17—C12	−59.9 (2)
C7—C8—C10—C1	59.9 (2)	C18—C19—C20—C14	−60.4 (2)
C9—C3—C4—C5	−59.7 (2)	C18—C19—C21—C12	60.7 (2)
C9—C8—C10—C1	−60.1 (2)	C20—C14—C15—C16	−59.8 (2)
C10—C1—C2—C3	−59.1 (2)	C20—C19—C21—C12	−59.1 (2)
C10—C1—C6—C5	59.1 (2)	C21—C12—C13—C14	−59.4 (2)
C10—C1—C11—N1	176.65 (16)	C21—C12—C17—C16	58.9 (2)
C10—C8—C9—C3	59.9 (2)	C21—C12—C22—N2	−177.13 (16)
C11—C1—C2—C3	−176.61 (16)	C21—C19—C20—C14	59.0 (2)
C11—C1—C6—C5	176.84 (16)	C22—C12—C13—C14	−176.66 (16)
C11—C1—C10—C8	179.95 (15)	C22—C12—C17—C16	176.07 (15)
C23—O2—C24—C23^i^	57.1 (3)	C22—C12—C21—C19	179.66 (15)

Symmetry code: (i) −*x*+1, −*y*+1, −*z*+1.

### Hydrogen-bond geometry (Å, º)

**Table:**

*D*—H···*A*	*D*—H	H···*A*	*D*···*A*	*D*—H···*A*
O1—H1*D*···Cl2^ii^	0.82 (3)	2.48 (3)	3.295 (2)	175 (3)
O1—H1*E*···Cl1	0.91 (4)	2.36 (4)	3.265 (2)	180 (3)
N1—H1*A*···Cl2^iii^	0.85 (3)	2.33 (3)	3.161 (2)	163 (2)
N1—H1*B*···O2	0.89 (3)	2.10 (3)	2.867 (3)	144 (2)
N1—H1*C*···Cl1	0.98 (3)	2.19 (3)	3.152 (2)	166 (2)
N2—H2*C*···Cl2^ii^	0.86 (2)	2.31 (3)	3.166 (2)	172 (2)
N2—H2*D*···Cl1	0.86 (3)	2.48 (3)	3.171 (2)	138 (2)
N2—H2*E*···Cl2	0.90 (3)	2.30 (3)	3.181 (2)	166 (2)

Symmetry codes: (ii) −*x*+2, −*y*, −*z*+1; (iii) *x*−1, *y*, *z*.
